# The Pseudokinase Domain of *Saccharomyces cerevisiae* Tra1 Is Required for Nuclear Localization and Incorporation into the SAGA and NuA4 Complexes

**DOI:** 10.1534/g3.118.200288

**Published:** 2018-04-13

**Authors:** Matthew D. Berg, Julie Genereaux, Jim Karagiannis, Christopher J. Brandl

**Affiliations:** * Department of Biochemistry, Schulich School of Medicine & Dentistry, Western University, London, Ontario, Canada N6A5C1; † Department of Biology, Western University, London, Ontario, Canada N6A5B7

**Keywords:** Tra1, SAGA complex, NuA4 complex, gene expression, phosphoinositide 3-kinase (PI3K) domain

## Abstract

Tra1 is an essential component of the SAGA/SLIK and NuA4 complexes in *S. cerevisiae*, recruiting these co-activator complexes to specific promoters. As a PIKK family member, Tra1 is characterized by a C-terminal phosphoinositide 3-kinase (PI3K) domain. Unlike other PIKK family members (*e.g.*, Tor1, Tor2, Mec1, Tel1), Tra1 has no demonstrable kinase activity. We identified three conserved arginine residues in Tra1 that reside proximal or within the cleft between the N- and C-terminal subdomains of the PI3K domain. To establish a function for Tra1’s PI3K domain and specifically the cleft region, we characterized a *tra1* allele where these three arginine residues are mutated to glutamine. The half-life of the Tra1${}_{\text{Q}3}$ protein is reduced but its steady state level is maintained at near wild-type levels by a transcriptional feedback mechanism. The *tra1*${}_{Q3}$ allele results in slow growth under stress and alters the expression of genes also regulated by other components of the SAGA complex. Tra1${}_{\text{Q}3}$ is less efficiently transported to the nucleus than the wild-type protein. Likely related to this, Tra1${}_{\text{Q}3}$ associates poorly with SAGA/SLIK and NuA4. The ratio of Spt7^SLIK^ to Spt7^SAGA^ increases in the *tra1*${}_{Q3}$ strain and truncated forms of Spt20 become apparent upon isolation of SAGA/SLIK. Intragenic suppressor mutations of *tra1*${}_{Q3}$ map to the cleft region further emphasizing its importance. We propose that the PI3K domain of Tra1 is directly or indirectly important for incorporating Tra1 into SAGA and NuA4 and thus the biosynthesis and/or stability of the intact complexes.

Tra1 is an essential 3744 residue co-activator protein in *Saccharomyces cerevisiae* that is a component of the multi-subunit SAGA (Spt-Ada-Gcn5 acetyltransferase) and NuA4 (nucleosome acetyltransferase of H4) complexes (Grant *et al.* 1998; Saleh *et al.* 1998; Allard *et al.* 1999). The mammalian ortholog of Tra1, TRRAP, is similarly found in the human SAGA (hSAGA) complex and the NuA4-related Tip60 complex (McMahon *et al.* 1998; Ikura *et al.* 2000). These complexes regulate gene expression as well as DNA repair through their ability to modify histones (reviewed in Nagy and Tora (2007) and Lu *et al.* (2009)). Also of note, both SAGA and NuA4 acetylate proteins other than histones (Mitchell *et al.* 2011, 2013; Law *et al.* 2014). For NuA4, these targets include the septin proteins, linking NuA4 to cytokinesis (Mitchell *et al.* 2011).

The SAGA complex is arranged into distinct modules, with Tra1 occupying its own module (Wu *et al.* 2004; Lee *et al.* 2011; Han *et al.* 2014; Setiaputra *et al.* 2015; Diaz-Santin *et al.* 2017). Other modules include the Ada module containing the lysine acetyltransferase Gcn5 (Grant *et al.* 1997; Govind *et al.* 2007) and the Ubp8 module which, among other functions, deubiquitinylates histone H2B (Henry *et al.* 2003). Microarray-based transcriptome analysis in strains deleted for SAGA components reveal that SAGA regulates approximately 10% of the yeast genome, particularly genes involved in stress response (Huisinga and Pugh 2004). However, mapping of SAGA localization across the genome and altered histone acetylation and deubiquitination patterns in strains lacking Gcn5 or Ubp8 indicate that SAGA likely plays a more global role in transcription (Bonnet *et al.* 2014; Baptista *et al.* 2017). The only known role for Tra1 in SAGA is to interact with transcriptional activators to recruit the complex to specific promoters (Brown *et al.* 2001; Bhaumik *et al.* 2004; Reeves and Hahn 2005; Knutson and Hahn 2011; Lin *et al.* 2012). Despite its large size, Tra1 is not simply a scaffold for SAGA (Helmlinger *et al.* 2011). Rather Spt7, Spt20 and Ada1 stabilize SAGA (Sterner *et al.* 1999; Wu and Winston 2002), while the TAF proteins form a scaffold around which the modules of SAGA are arranged (Wu *et al.* 2004; Han *et al.* 2014). Yeast also have SLIK, a SAGA-like complex that is identical to SAGA, except it lacks Spt8, has a C-terminally truncated form of Spt7 and may contain Rtg2 (Pray-Grant *et al.* 2002; Sterner *et al.* 2002; Wu and Winston 2002). The role of SLIK increases in the transcriptional response to stress (Belotserkovskaya *et al.* 2000; Pray-Grant *et al.* 2002; Sterner *et al.* 2002; Spedale *et al.* 2010).

Tra1 is essential due to its functions in NuA4 (Helmlinger *et al.* 2011). NuA4, like SAGA, is modular (Chittuluru *et al.* 2011) with its catalytic subunit being the lysine acetyltransferase Esa1 (Allard *et al.* 1999; Clarke *et al.* 1999). Again Tra1 comprises its own module with its only defined role being to interact with transcriptional activators thus recruiting the complex to specific promoters (Chittuluru *et al.* 2011).

Tra1/TRRAP is a member of the phosphoinositide 3-kinase-related kinase (PIKK) protein family. Other family members are ataxia telangiectasia mutated (ATM/Tel1), ATM and Rad3-related (ATR/Mec1), DNA-dependent protein kinase catalytic subunit (DNA-PKcs), mechanistic target of rapamycin (mTOR/Tor1,Tor2) and suppressor with morphological effect on genitalia family member (SMG-1) (Lempiäinen and Halazonetis 2009). These proteins share four principle domains: an N-terminal HEAT region, followed by FAT, PI3K, and FATC domains (Lempiäinen and Halazonetis 2009; Knutson and Hahn 2011; Baretić *et al.* 2017; Sibanda *et al.* 2017; Diaz-Santin *et al.* 2017). The helical HEAT repeats comprise approximately half of the molecule. It is this region of Tra1 that interacts with activator proteins (Brown *et al.* 2001; Bhaumik *et al.* 2004; Lin *et al.* 2012). C-terminal to the HEAT repeats is the helical FAT domain, which forms a cap wrapping the N-terminal portion of the PI3K domain (Yang *et al.* 2013; Sibanda *et al.* 2010). After the FAT domain, the PIKK proteins have an ∼100 residue FRB domain, which is the FKBP12 and rapamycin binding domain in mTOR (Chen *et al.* 1995). Toward their C-terminal, the PIKK proteins share a highly conserved kinase domain similar to the phosphoinositide 3-kinases (PI3K). Typical of all kinases, the PI3K domain consists of N- and C-terminal subdomains connected by a hinge region that forms the ATP-binding pocket in the catalytically active PIKK family members. The flexibility of the hinge region is responsible for the orientation of the N- and C-lobes. In most kinases, including the PIKK PI3K domains, the N-lobe is composed mainly of *β*-strands and undergoes significant rearrangement during catalysis, whereas the C-lobe is composed of more rigid *α*-helices (reviewed in Kung and Jura (2016)). Interestingly, in all PIKK proteins except Tra1/TRRAP (McMahon *et al.* 1998; Saleh *et al.* 1998; Mutiu *et al.* 2007), the PI3K domain phosphorylates downstream targets to facilitate signaling (Lovejoy and Cortez 2009).

The cleft formed between the N- and C-lobes binds ATP in the kinase members of the PIKK family (Rivera-Calzada *et al.* 2005; Yang *et al.* 2013). The kinase domain of Tra1 lacks several residues within the cleft required for catalysis, including a lysine that interacts with the *α*-phosphate of ATP and crosslinks to the furan ring of wortmanin (Wymann *et al.* 1996), and a tryptophan found in the hinge linking the subdomains that stacks with the adenine purine ring. In addition, Tra1 lacks the catalytic aspartic acid residue of the HRD motif and the Mg^2+^ binding DFG motif (reviewed in Boudeau *et al.* (2006)). Currently, no function has been ascribed to the PI3K domain of Tra1. Crosslinking experiments of SAGA components show that the PI3K domain of Tra1 only crosslinks to the FAT domain, not with other SAGA components (Han *et al.* 2014).

At the extreme C-terminus the PIKK proteins share a FATC domain. In all cases the FATC domain ends with two hydrophobic residues. Additions or deletions to the terminus result in loss of function (Hoke *et al.* 2010; DaSilva *et al.* 2013). In mTOR, the FATC domain integrates with the PI3K domain forming part of the substrate binding pocket. Mutations in the FATC domain of Tra1 destabilize the protein and are suppressed by mutations in the PIKK co-chaperone protein Tti2 (Genereaux *et al.* 2012). In mTOR, the FATC domain wraps back toward the kinase domain and packs with the activation loop, possibly stabilizing the structure and providing contacts for substrate binding (Yang *et al.* 2013).

Though not likely a kinase, mutational analyses indicate that the PI3K domain is required for Tra1 function. Previously, we examined a set of alleles in the context of plasmid copies of *tra1* that altered residues found within both the N- and C-lobes. Of these two single mutants, R${}_{3456}$A and R${}_{3567}$A, and six triple alanine mutants at WRR${}_{3317}$, DIE${}_{3351}$, RFL${}_{3374}$, FRK${}_{3544}$, PFR${}_{3621}$ and RDE${}_{3668}$ resulted in loss of viability (Mutiu *et al.* 2007). Similarly, Knutson and Hahn (2011) identified two essential surface residues, R3650 in the C-lobe and K3423 within the N-lobe. Other alleles, SRR${}_{3413}$AAA, P${}_{3408}$A and S${}_{3463}$A resulted in transcriptional changes and slow growth in stress conditions (Mutiu *et al.* 2007). We find that the phenotype of these alleles is less severe when they are integrated. Our goal was to identify a *tra1* allele that when integrated, produced a slow growth phenotype and could be characterized biochemically and genetically to elucidate the function of the PI3K domain of Tra1.

By analogy to other PIKK family members, we predicted that the PI3K domain of Tra1 has a regulatory role. If so, residues within the “ATP-binding” cleft would be required for Tra1 function. Using sequence alignments and the mTOR PI3K crystal structure (Yang *et al.* 2013), we identified three arginine residues proximal to the PI3K ATP-binding cleft that when mutated to glutamine compromise cell growth and result in transcriptional defects. The altered protein, which we call Tra1${}_{\text{Q}3}$, is expressed at near wild-type levels but its half-life is reduced. A transcriptional feedback mechanism sensitive to functional Tra1 helps maintain cellular levels of Tra1${}_{\text{Q}3}$. Tra1${}_{\text{Q}3}$ is less efficiently transported to the nucleus and its incorporation into the SAGA and NuA4 complexes is reduced. In the *tra1${}_{\mathrm{Q3}}$* strain, Spt20 is prone to proteolysis and there is an increased level of the truncated SLIK-form of Spt7. We also identify and characterize intragenic suppressors within the kinase cleft that restore localization defects of Tra1${}_{\text{Q}3}$. Together these results emphasize the importance of the PI3K domain of Tra1 and suggest a role for the domain in the incorporation of Tra1 into the SAGA and NuA4 complexes.

## Materials and Methods

### Yeast strains

Yeast strains used in this study are derivatives of the haploid BY4741 and BY4742 and the diploid BY4743 (Winzeler and Davis (1997); Table S1). CY4353 (*TRA1-HIS3*) is described in Hoke *et al.* (2010). CY4398 containing *TRA1/URA3-Flag*^5^*-TRA1* is described in Genereaux *et al.* (2012). The non-tagged *TRA1* allele of CY4398 was gene replaced with *tra1${}_{\text{Q}3}$* along with a *HIS3* allele at the downstream *Bst*BI site in *YHR100C/GEP4* to create CY6577. CY6577 was sporulated to generate MAT*α* (CY6582) and MATa (CY6586) strains. CY6554 and CY6517 were created identically and contain *tra1-R3456Q* and *tra1-R3389Q*, *R3390Q* respectively. All haploid strains contain YCplac111-*DED1pr-YHR100C* or YCplac33-*DED1pr-YHR100C*. *tra1*${}_{Q3}$ allele was integrated into the Flag-tagged allele of CY4398 and sporulated to create the haploid *URA3-Flag*^5^*-tra1*${}_{Q3}$*-HIS3* (CY6605). *tra1*${}_{Q3}$ allele was integrated into the haploid *URA3-eGFP-TRA1* strain CY5998 (Genereaux *et al.* 2012) to create CY7341. CY8071 (*TRA1/tra1-R3389A*, *R3390A*, *R3456A*) was created by gene replacement with *Sph*I-*Sac*I fragment of pCB4314. TAP-tagged *ADA2* (YSC1178-202230655) and *EPL1* (YSC1178-202230938) were purchased from Open Biosystems. These strains were crossed with CY6582 and sporulated to obtain strains with *tra1*${}_{Q3}$ and TAP-*ADA2* (CY7042) or TAP-*EPL1* (CY7253). Strains with suppressor mutations were isolated using the selection method described below. The *tra1* allele of CY6902, CY6909, CY6940 and CY6715 was tagged with eGFP by integrating an *Sph*I-*Xba*I fragment of pCB2301 or with a Flag-tag by integrating an *Sph*I-*Xba*I fragment of pCB2416 (as described in Genereaux *et al.* (2012)).

### DNA molecules

To create a *tra1-R3456Q* integrating vector, a *Bam*HI-*Eco*RV fragment of *TRA1* PI3K domain containing the mutation was purchased from Life Technologies (Figure S1A). This molecule was cloned into the integrating vector pCB2147 described by Hoke *et al.* (2010) to create pCB2527. The integrating *tra1-R3389Q*, *R3390Q* vector was similarly made from an *Xba*I-*Bam*HI fragment containing the mutation (Figure S1B) and cloned into the integrating vector to create pCB2484. These alleles were integrated as *Sph*I-*Sac*I fragments. The *tra1${}_{\text{Q}3}$* allele was created by cloning the *Sph*I-*Bam*HI fragment from pCB2484 into pCB2527. To create a *tra1-R3389A*, *R3390A*, *R3456A* integrating molecule, the R3389A, R3390A mutations were introduced into the *tra1-R3456A* plasmid (Mutiu *et al.* 2007) by two step PCR using outside primers 4333-1/4225-6 with VF8129/VF8130 (Table S2). The PCR fragment was cut *Xba*I-*Eco*RV and cloned into the Tra1 integrating vector to create pCB4134. Myc^9^-tagged *SPT7* was expressed from the *DED1* promoter in YCplac111, a *LEU2* centromeric plasmid, by inserting the *Not*I-*Eco*RI fragment from YCp88-myc-*SPT7* (Hoke *et al.* (2007)) downstream of a *DED1* promoter-myc^9^ cassette (Hoke *et al.* 2010). C-terminally myc^9^-tagged *SPT20* was expressed by inserting a *Bam*HI-*Hin*dIII fragment amplified from genomic DNA using oligonucleotides UI7081 and 2039-2 and the myc^9^-tag as a *Hin*dIII-*Sac*I fragment from pCB2890 (Hoffman *et al.* 2016) into YCplac33. eGFP-Spt7 was made by moving *SPT7* as a *Not*I-*Eco*RI fragment from the myc^9^-tagged vector into YCplac33-*DED1pr-EGFP* vector previously described in Hoke *et al.* (2008b). LacZ reporter constructs were cloned as *his3-lacZ* fusions into the *LEU2* centromeric plasmid YCplac87 (Brandl *et al.* 1993). The SRE-lacZ, *PHO5*-lacZ, *GAL10*-lacZ and *ARG1*-lacZ fusion constructs in YCplac87 have been described (Martens *et al.* 1996; Ricci *et al.* 2002; Hoke *et al.* 2010). *TRA1* promoter sequences -975 to -9 relative to the translational start and *NCW2* promoter sequences -704 to +64 were engineered by PCR as *Bam*HI-*Hin*dIII fragments using oligonucleotides listed in Table S2. The *HTA2-mCherry* construct was made by amplifying *HTA2* from genomic DNA using VJ3060/VJ3061 and *mCherry* from a plasmid (Addgene #20972; Nam and Benezra (2009)) using VJ2726/VJ2727. The amplified fragments were cloned into pRS41K (Taxis and Knop 2006).

### Growth assays

Growth was compared on yeast peptone (YP) media containing 2% glucose (YPD) after 2-5 days at 30° unless otherwise stated. Stress sensitivity was tested on YPD plates containing either 6% ethanol, 8 or 6 *μ*g/mL Calcofluor White (Sigma-Aldrich), 1 M sodium chloride (EMD Chemicals Inc.), 100 mM calcium chloride (BDH Chemicals), 15 mM caffeine (Sigma-Aldrich) or 0.03% methyl methanesulfonate (MMS; Sigma-Aldrich). Growth was also assayed on YPD plates depleted of phosphate (Han *et al.* 1988) or on medium lacking inositol (BD Difco Inositol Assay Medium). Strains were grown to stationary phase and spotted in ten-fold serial dilutions.

### Half-life

Yeast strains were grown to stationary phase in media lacking leucine, diluted 1:25 in YPD and grown to an OD${}_{600}$ of 2.0 before adding 35 *μ*g/mL cycloheximide. Five mL of cells were harvested before adding cycloheximide and at two, four, six and eight hours thereafter. Cells were lysed with glass beads in the presence of protease inhibitors (1 mM phenylmethylsulfonyl fluoride, 5 mg/mL pepstatin, 1 mM benzamidine, 50 mg/mL trypsin inhibitor and 5 mg/mL leupeptin), the lysates separated by SDS-PAGE and western blotted as described below. The standard deviation of three biological replicates is reported.

### ***β***-galactosidase assay

Yeast strains containing lacZ reporter plasmids were grown to stationary phase, washed three times with sterile water, diluted 10-fold in media to induce the reporter and grown eight hours. The SRE-*lacZ* reporter was induced in YPD containing 6% ethanol; *PHO5-lacZ* in medium depleted of phosphate; *GAL10-lacZ* in YP medium containing 2% galactose; and *ARG1-lacZ*, *NCW2-lacZ* and *TRA1-lacZ* in YPD medium. *β*-galactosidase units were determined using o-Nitrophenyl-β-galactoside as substrate and values were normalized to cell densities.

### Quantitative RT-PCR

Cells were grown to stationary phase, diluted 10-fold into YPD and grown eight hours. Cells were harvested and RNA isolated using the MasterPure Yeast RNA Purification Kit (Epicentre). cDNA was synthesized from 1 *μ*g of RNA using the qScript Flex cDNA kit (QuantaBio). Real-time PCR was carried out using a Bio-Rad CFX Connect Real-Time PCR Detection System with PerfeCTa SYBR Green FastMix (QuantaBio). Reactions were performed in triplicate using the *TRA1* primer pair Tra1F-qPCR/Tra1R-qPCR (Table S2). Transcript levels were normalized to U3 RNA (U3F-qPCR/U3R-qPCR). Data were analyzed using the Bio-Rad CFX Manager software, version 3.1 (Bio-Rad).

### Fluorescence microscopy

Yeast strains expressing eGFP-Spt7 or eGFP fusions of Tra1 PI3K alleles were prepared and imaged as described in Hoffman *et al.* (2016). Strains were grown to stationary phase in minimal media lacking uracil, diluted 1:20 and grown 5 hr before 2.5 *μ*g/mL 4,6-Diamidino-2-Phenylindole, Dihydrochloride (DAPI; Sigma-Aldrich) was added. Cells were imaged using a Zeiss Axioskop 2 confocal microscope driven by Image J 1.41 software (NIH) and a Scion CFW Monochrome CCD Firewire camera (Scion, Fredrick MD) with bright field, DAPI, GFP and Texas Red filter sets. Exposure time and gain settings were left constant across all images captured. Cell measurements were taken with ImageJ 1.50i using the *HTA2-mCherry* construct to mark the nucleus. Whole cells were identified using the bright field images.

### Cellular fractionation

Fractionation was performed as described in Keogh *et al.* (2006). Briefly, 50 mL of cells were grown to an OD${}_{600}$ of ∼1.0 in YPD, collected by centrifugation and washed in ddH${}_{2}$O followed by SB (1 M Sorbitol, 20 mM Tris-Cl pH 7.4). Cells were resuspended in 1 mL SB and rotated at room temperature with 125 *μ*g Zymolase 20T to make spheroplasts. Spheroplasts were washed with SB and resuspended in 500 *μ*L EBX (20 mM Tris-Cl pH 7.4, 100 mM NaCl, 0.25% Triton X-100, 15 mM *β*-mercaptoethanol + protease inhibitors). Triton X-100 was added to 0.5% to lyse the outer membrane and the lysate was layered over 1 mL NIB (20 mM Tris-Cl pH 7.4, 100 mM NaCl, 1.2 M Sucrose, 15 mM *β*-mercaptoethanol + protease inhibitors) and centrifuged at 12K for 15 min at 4°. The upper layer was taken as the cytoplasmic fraction. The nuclear pellet was re-suspended in 500 *μ*L EBX and Triton X-100 was added to 1% final to lyse the nuclear membrane. Triton X-100 permeabilized fraction were spotted onto a PVDF membrane and blotted with anti-Flag antibody to detect Tra1, while 3X SDS-PAGE loading buffer (188 mM Tris-Cl, 3% SDS, 30% glycerol, 0.01% bromophenol blue, 180 mM DTT) was added to both fractions, separated on an 18% SDS-PAGE gel and used in western blotting for histone H3.

### Protein extracts and immunoprecipitations

Yeast strains were grown in YPD media to an OD${}_{600}$ of ∼2.0. Cells were harvested and ground in liquid nitrogen as previously described (Saleh *et al.* 1997). Immunoprecipitations were performed at 4°. Ground lysates were suspended in IPP150 (10 mM Tris-HCl pH 8.0, 150 mM NaCl) containing protease inhibitors. Lysates were incubated with IgG beads (Sigma-Aldrich) for 2 hr. Beads were washed three times with IPP150 containing 0.1% NP-40. Protein was cleaved with TEV protease in 10 mM Tris-HCl pH 8.0, 150 mM NaCl, 0.1% NP-40, 0.5 mM EDTA, 1 mM DTT for 2.5 hr. Calmodulin binding buffer (10 mM Tris-HCl pH 8.0, 150 mM NaCl, 10 mM *β*-mercaptoethanol, 1 mM Mg-acetate, 1 mM imidazole, 2 mM CaCl${}_{2}$ and 0.1% NP-40) was added to the eluted protein and incubated with calmodulin beads (Agilent Technologies) for an hour. The column was washed 3x in calmodulin binding buffer then protein was eluted in five 200 *μ*L fractions with 10 mM Tris-HCl pH 8.0, 150 mM NaCl, 10 mM *β*-mercaptoethanol, 1 mM Mg-acetate, 1 mM imidazol, 2 mM EGTA, 0.1% NP-40.

### Western blotting

Yeast strains were lysed either using glass beads or by grinding in liquid nitrogen (Saleh *et al.* 1997). After separating proteins using SDS-PAGE, protein was transferred to PVDF membrane and blotted using anti-Flag (M2; Sigma-Aldrich) or anti-myc (9E10; Sigma-Aldrich) antibodies as described by Mutiu *et al.* (2007) or Hoke *et al.* (2010). Histone H3 was detected using anti-H3 (ab1791, Abcam). Band intensity was measured using ImageJ 1.50i.

### Mass spectrometry

Samples were prepared at the Functional Proteomics Facility (University of Western Ontario, Department of Biochemistry, http://www.uwo.ca/biochem/fpf/). Proteins separated on a polyacrylamide gel stained with Coomassie Brilliant Blue were selected using the Ettan Spot Picker robotic system. Gel pieces were destained in 50 mM ammonium bicarbonate and 50% acetonitrile, reduced in 10 mM dithiothreitol (DTT), alkylated using 55 mM iodoacetamide (IAA) and digested with trypsin (prepared in 50 mM ammonium bicarbonate, pH 8). A Waters MassPREP Station (PerkinElmer) was used for in-gel digestion. Peptides were extracted in 1% formic acid and 2% acetonitrile, then lyophilized. Prior to mass spectrometry analysis, dried peptide samples were re-dissolved in 10% acetonitrile and 0.1% trifluoroacetic acid.

MALDI matrix, *α*-cyano-4-hydroxycinnamic acid (CHCA), was prepared at 5 mg/mL in 6 mM ammonium phosphate monobasic, 50% acetonitrile, 0.1% trifluoroacetic acid and mixed with the sample at 1:1 ratio (v/v). Mass spectrometry was performed using the AB Sciex 5800 TOF/TOF System, MALDI TOF TOF (Framingham, MA, USA). Data acquisition and data processing were done using a TOF TOF Series Explorer and Data Explorer (both from AB Sciex) in reflectron positive mode. The instrument is equipped with a 349 nm OptiBeam On-Axis laser with a pulse rate of 400 Hz. Reflectron mode was externally calibrated at 50 ppm mass tolerance and internally at 10 ppm. Each mass spectrum was collected as a sum of 500 shots. Data files have been included in a supplemental file. Data files were searched using MASCOT (Matrix Science) against the NCBI database allowing one missed cleavage from trypsin and using cysteine carbamidomethyl as a fixed modification, oxidation of methionine as a variable modification and a peptide mass tolerance of 30 ppm.

### Suppressor selection and identification

CY6582 containing YCplac111-*DED1pr-YHR100C* was grown to stationary phase in media lacking leucine. 50 *μ*L (∼10 million cells) were plated on YPD plates containing 6% ethanol and either UV irradiated at a wavelength of 302 nm for 10 sec, with a survival rate of ∼10%, or simply incubated at 30°. An independent set of cultures was also treated with sodium azide following the protocol outlined by Silhánková *et al.* (1979). Colonies that grew after 4 days at 30° were isolated under non-selective conditions and their growth reanalyzed on YPD containing 6% ethanol. Isolated strains were crossed with BY4741 and sporulated to determine linkage of the suppressor with *tra1*${}_{Q3}$. The PI3K domain of the gene from the suppressor strains was amplified using oligonucleotides 4333-1 and 4225-6 and sequenced using 4333-1 as the primer.

This is stated twice (also below).

### Data availability

The authors affirm that all data necessary for confirming the conclusions of this article are represented fully within the article and its tables and figures. All yeast strains and plasmids are available upon request. Supplemental material available at Figshare: https://doi.org/10.25387/g3.6100331.

## Results

### A tra1 allele with a compromised PI3K domain

**Figure 1:**
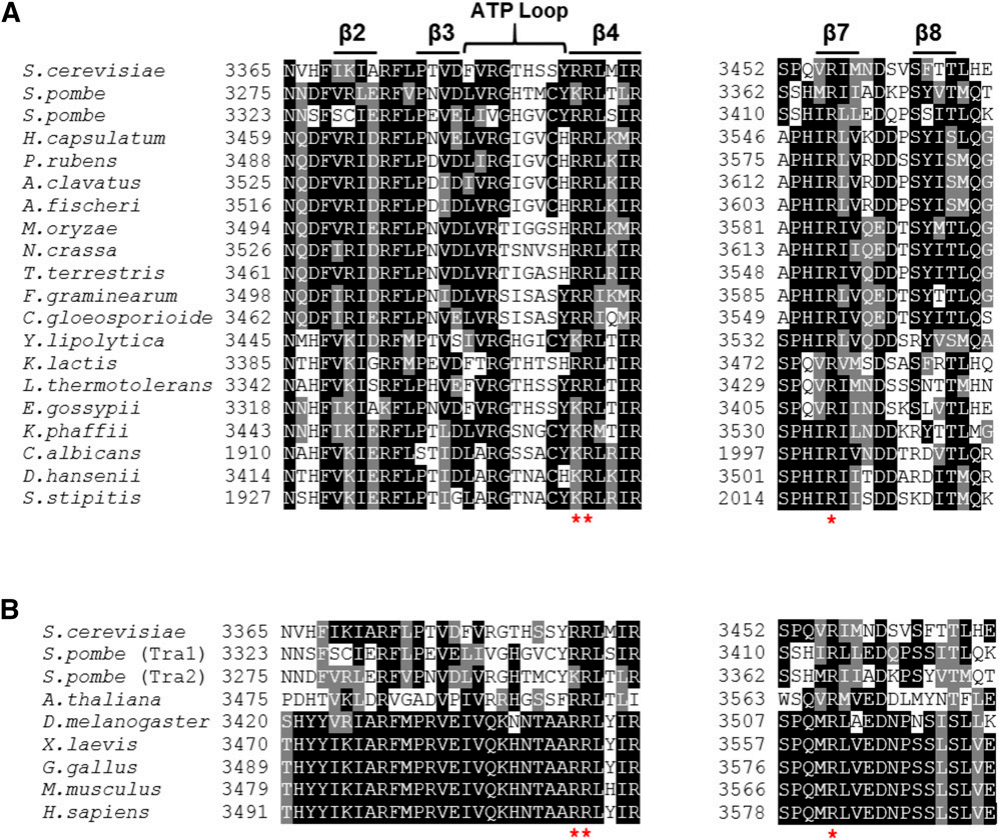
Sequence alignment of Tra1 homologs. (A) Sequence alignment of the PI3K domain region near the ATP binding cleft of various fungal Tra1 homologs. Three conserved arginine residues (R3389, R3390 and R3456) within the PI3K domain are marked with *. Alignments were performed using the defaults of the MUSCLE tool [http://www.ebi.ac.uk/Tools/msa/muscle/]. (B) Sequence alignment of the region surrounding the conserved arginine residues identified above from a broader range of Tra1 homologs. The arginine residues are marked by * and alignments were performed as above.

**Figure 2:**
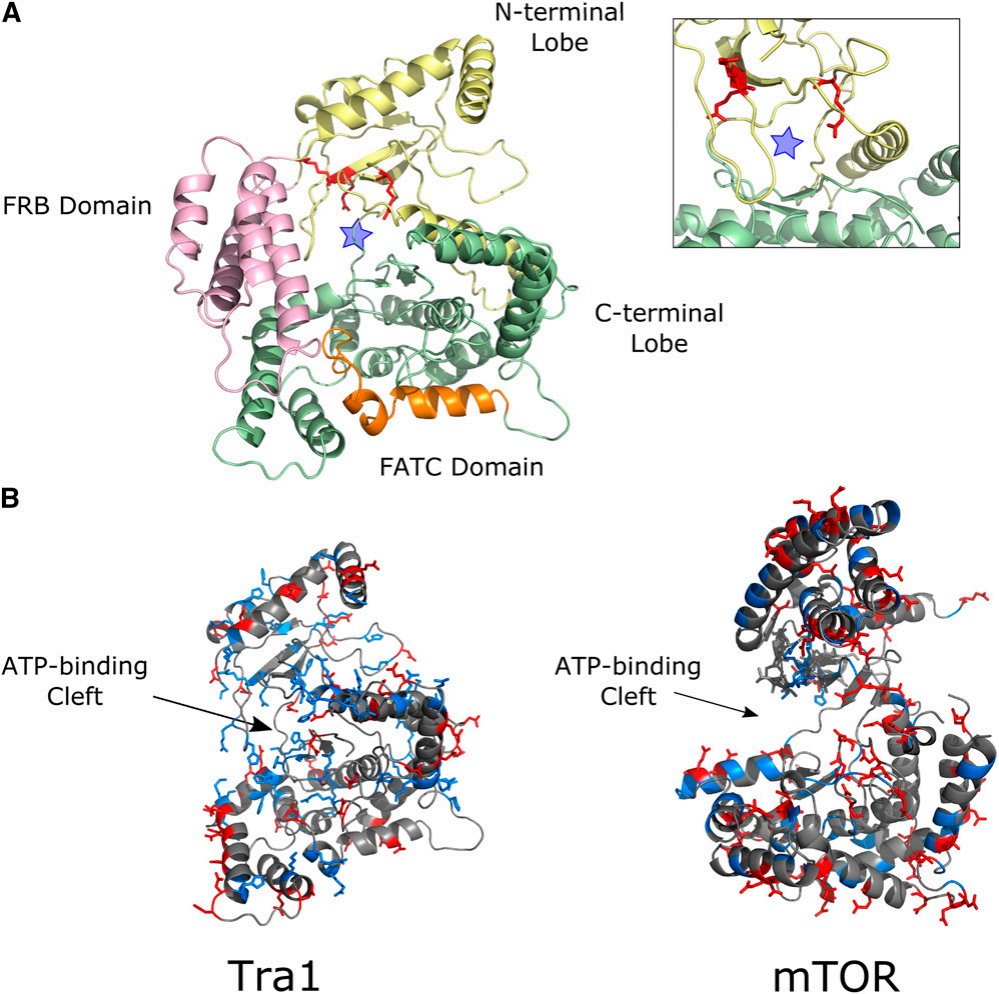
Structural prediction of the Tra1 PI3K domain. (A) Arginines at positions 3389, 3390 and 3456, identified from the alignments, were mapped onto the cryo-EM structure of Tra1 and colored red (Diaz-Santin *et al.* (2017); PDB: 5OJS; residues 3200-3744 are shown). The “ATP-binding” cleft is marked by a blue star. (B) The distribution of positive and negatively charged residues in the Tra1 PI3K domain (as above) was compared to the distribution in the mTOR crystal structure (Yang *et al.* (2013); PDB: 4JSV; residue 2000 - 2549). PyMOL (https://pymol.org/2/) was used to color basic residues blue and acidic residues red. The FRB domain of Tra1 (residues 3240 - 3330) has been removed to better visualize the kinase cleft. Both molecules are oriented using the ATP-binding cleft, with the N-terminal lobe of the kinase at the top of the representation.

Our goal was to use molecular genetic approaches to establish a function for the PI3K domain of Tra1. The analysis required identifying an allele of *tra1* that compromised growth but would still support viability. We predicted that residues proximal to the ATP-binding cleft found in the kinase members of the PIKK family may be involved in an essential interaction. To identify candidate residues, we aligned fungal Tra1 homologs (Figure 1A). We noted three conserved arginine residues, at positions 3389, 3390 and 3456 whose positively charged side chain suggested a surface location. As shown in Figure 1B, these residues are also conserved in alignments of a broader range of Tra1 homologs. When aligned with the corresponding sequences of Tor1, Tor2, Mec1 and Tel1 it was apparent that only the positive charge at position 3389 is conserved across the family suggesting that the arginines may have a unique function in Tra1 (Figure S2). We mapped the location of arginine 3389, 3390 and 3456 on the recent cryo-EM structure of Tra1 (Diaz-Santin *et al.* 2017). The three conserved arginine residues fall within or near the ATP-binding cleft (Figure 2A), though their exact orientation is difficult to predict at the resolution of the structure (3.7Å). As shown in Figure 2B, the ATP-binding cleft of Tra1 differs from that of mTOR (Yang *et al.* 2013) in that it has more surface exposed positively charged residues and is smaller. In addition, the sequence corresponding to the FRB domain originally defined in mTOR, blocks the Tra1 cleft region (seen in Figure 2A).

**Figure 3:**
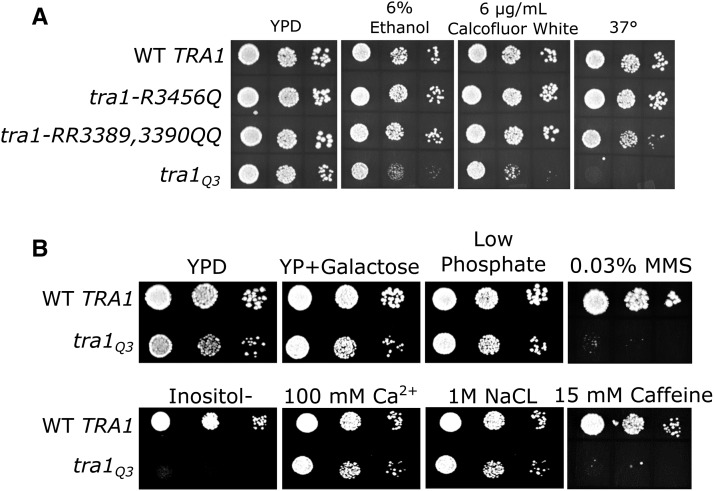
Growth of yeast strains containing integrated copies of *tra1* with mutations of R3389, R3390 and/or R3456 to glutamine. (A) *TRA1* (CY4353), *tra1-R3456Q* (CY6554), *tra1-R3389Q*, *R3390Q* (CY6517) and *tra1-R3389Q*, *R3390Q*, *R3456Q* (*tra1${}_{\mathrm{Q3}}$*; CY6582) were grown to saturation in media lacking leucine and spotted in 10-fold serial dilutions onto a YPD plate at 30° or onto a YPD plate containing 6% ethanol or 6 *μ*g/ml Calcoflour white or onto a YPD plate at 37°. Strains were incubated for 3-4 days. (B) *TRA1* (CY4353) and *tra1${}_{\mathrm{Q3}}$* (CY6582) were grown to saturation in media lacking leucine then spotted in 10-fold serial dilutions onto YPD media or YPD media depleted of phosphate, YPD media containing MMS or caffeine, minimal media lacking inositol, minimal media containing calcium or salt or a YP plate containing 2% galactose. Strains were incubated for 2-5 days at 30°.

We created three *tra1* alleles, *tra1-R3456Q*, *tra1-R3389Q,R3390Q* and *tra1${}_{\text{Q}3}$* (R3389Q, R3390Q, R3456Q), to investigate the importance of the arginine residues. The alleles were integrated into a diploid yeast strain where they are expressed from their native promoter. Haploid strains with the mutant alleles as the sole copy of *TRA1* were obtained by sporulation. Strains were examined for growth on rich medium at 30°, and medium containing 6% ethanol or 6 *μ*g/ml Calcoflour white, and at 37°, which result in slow growth in strains containing deletions of SAGA components (Mutiu *et al.* 2007; Hoke *et al.* 2010; Genereaux *et al.* 2012). As shown in Figure 3A, the strain containing *tra1-R3456Q* grew at a near wild-type rate in these conditions. The *tra1-R3389Q*, *R3390Q* double mutant strain grew at a slightly reduced rate at 37°, but was not sensitive to ethanol or Calcofluor white. The triple mutant, *tra1*${}_{Q3}$, resulted in slow growth on rich media and severe slow growth on media containing 6% ethanol or Calcofluor white. We also analyzed growth of the *tra1*${}_{Q3}$ strain under other conditions of stress (Figure 3B). Severe slow growth was observed on media lacking inositol, a phenotype characteristic of defects in Spt7 (Gansheroff *et al.* 1995) and on media containing caffeine or the DNA damaging agent MMS. The latter two phenotypes are characteristic of strains with mutations in NuA4 components. Growth on media containing elevated sodium chloride or calcium chloride, galactose as a carbon source or media depleted of phosphate did not exacerbate the slow growth phenotype of the *tra1*${}_{Q3}$ strain relative to its growth on YPD. These results indicate the importance of the arginine residues for Tra1 function. We also constructed an allele where R3389, R3390 and R3456 were converted to alanine (*tra1${}_{A3}$*) and integrated it into a diploid strain. Upon sporulation, viability segregated 2:2 indicating that the triple alanine mutant eliminates Tra1 function.

**Figure 4:**
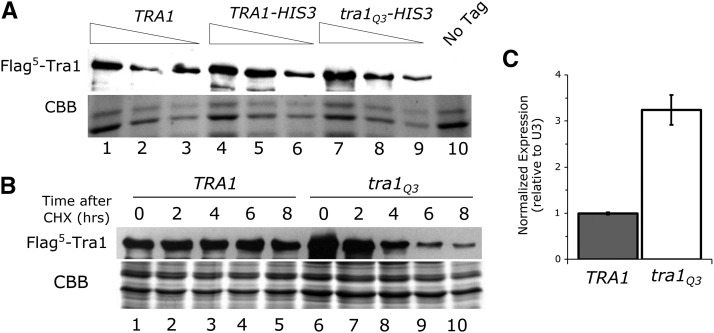
Steady state levels and half-life of Tra1${}_{\text{Q}3}$. (A) Yeast strains CY6808 (Flag^5^-tagged *TRA1*; lanes 1-3), CY5920 (Flag^5^-tagged *TRA1* with *HIS3* inserted downstream of the *TRA1* locus; lanes 4-6), CY6605 (Flag^5^-tagged *tra1${}_{\mathrm{Q3}}$* with *HIS3* inserted downstream of the *TRA1* locus; lanes 7-9) and BY4742 (No Tag, lane 10) were grown to stationary phase in media lacking leucine, diluted 1:20 in YPD and grown for eight hours. Protein lysates were prepared by bead lysis and 40 *μ*g, 20 *μ*g, and 10 *μ*g of total protein was loaded, except for the no tag control where 40 *μ*g was loaded. Extracts were separated by SDS-PAGE and blotted with anti-Flag antibody. The bottom of the gel was stained with Coomassie Brilliant Blue (CBB) as a loading control. (B) Yeast strains expressing Flag^5^-tagged Tra1${}_{\text{WT}}$ (lane 1-5; CY6808) or Tra1${}_{\text{Q}3}$ (lane 6-10; CY6605) were grown to saturation in media lacking leucine, diluted 1:25 and grown for 6 hr. Translation was inhibited with 35 *μ*g/ml cycloheximide. Cells were harvested at 0, 2, 4, 6 and 8 hr after cycloheximide addition and 40 *μ*g of total protein from each time point was separated by SDS PAGE and blotted with anti-Flag antibody. The bottom of the gel was stained with CBB as a loading control. (C) *TRA1* (CY4353) and *tra1${}_{\mathrm{Q3}}$* (CY6582) were grown to saturation in media lacking leucine, diluted 1:10 in YPD and grown for eight hours. Transcript levels of *TRA1* were measured and normalized to U3 (snR17a) as a loading control. Data represent mean values ± SD from three independent biological replicates.

To determine if the phenotypic changes caused by Tra1${}_{\text{Q}3}$ were due to its expression, we performed a western blot with the protein containing an N-terminal Flag-tag. As shown in Figure 4A, Tra1${}_{\text{Q}3}$ is present at a level slightly elevated relative to the wild-type protein. From four biological replicates, we calculate the ratio of Tra1${}_{\text{Q}3}$ to wild-type Tra1 to be 1.4 ± 0.4. To assess half-life of the protein, the strains were grown to mid-logarithmic phase before translation was inhibited with cycloheximide. Tra1 levels were examined after further incubation for 2, 4, 6 and 8 hr. The half-life of Tra1${}_{\text{Q}3}$ was 4.2 ± 1.6 hr, whereas the wild-type protein level did not decrease over eight hours (Figure 4B). When we examined the wild-type Tra1 levels at later time points after cycloheximide addition, we found the half-life to be 12 hr (Figure S3). The half-life of Tra1${}_{\text{Q}3}$ is thus reduced threefold.

The similar steady-state level but different half-life of wild-type Tra1 and Tra1${}_{\text{Q}3}$ suggested a feedback mechanism that increases expression of Tra1 when it is depleted. To test this further, we compared the steady state level of *tra1${}_{Q3}$* mRNA relative to wild-type *TRA1* mRNA in haploid strains CY6582 and CY4353, respectively, by RT-qPCR (Figure 4C). Supporting the feedback regulation of *TRA1*, the steady state level of the *tra1*${}_{Q3}$ mRNA was ∼3 fold higher than in the wild-type *TRA1* mRNA. We observed a similar increase in expression in the *tra1*${}_{Q3}$ strain relative to the wild-type when we assayed *β*-galactosidase activity from a *TRA1* promoter lacZ fusion (Figure S4). Deleting the SAGA components Gcn5, Spt20 or Ubp8 or the NuA4 component Eaf3 did not increase expression of this *TRA1*-lacZ fusion (Figure S4), suggesting that general loss of the functional complexes does not induce the feedback mechanism. Interestingly, *TRA1*-lacZ expression decreased in strains deleted for Spt20 or Ubp8, suggesting the deubiquitylation activity of SAGA plays a role in *TRA1* transcription.

### tra1${}_{\mathrm{Q3}}$ alters transcription from SAGA-dependent promoters and is synthetic lethal with many SAGA and NuA4 component genes

**Figure 5:**
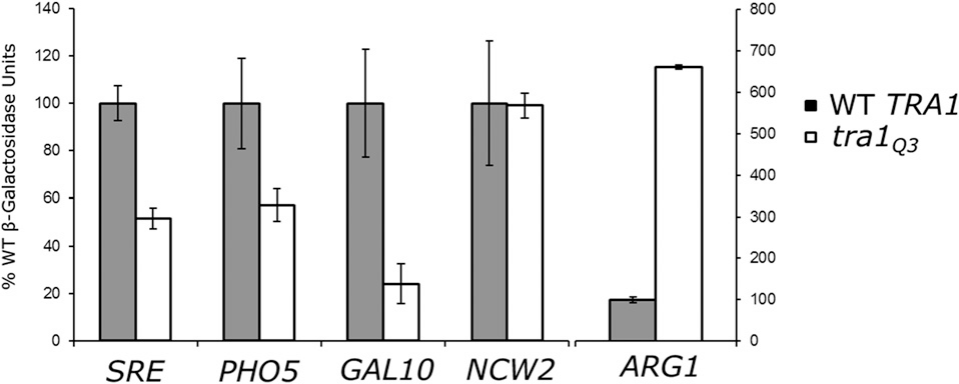
*tra1${}_{\mathrm{Q3}}$* affects expression from SAGA regulated promoters. *TRA1* (BY4741) and *tra1${}_{\mathrm{Q3}}$* (CY6635) were transformed with the indicated promoter-lacZ fusion molecules on a *LEU2* centromeric plasmid. Strains were grown to saturation in media lacking leucine before being diluted 1:10 in YPD with 6% ethanol for the SRE reporter, media lacking phosphate for the *PHO5* promoter, YP with galactose for the *GAL10* promoter or YPD for the *ARG1* and *NCW2* promoters and grown for eight hours. *β*-galactosidase activity is shown as the average of six replicates for the regulated promoters and three replicates for the *NCW2* promoter with the SD indicated by the error bars.

To evaluate the effect of *tra1*${}_{Q3}$ on transcription, we analyzed expression of four SAGA regulated promoters: *PHO5*, *GAL10*, *ARG1* and a synthetic promoter containing stress response elements (SRE). Of these, the *PHO5*, *GAL10* and *SRE* promoters require SAGA for activation (Bhaumik and Green 2002; Barbaric *et al.* 2003; Huisinga and Pugh 2004), whereas SAGA is required for the repression of *ARG1* in rich media (Ricci *et al.* 2002). Expression from the *NCW2* promoter was assayed to represent a promoter not regulated by SAGA or NuA4. LacZ promoter fusions were introduced into wild-type and *tra1*${}_{Q3}$ strains and *β*-galactosidase activity determined after growing cells for eight hours (Figure 5). Expression from the three promoters activated by SAGA (SRE, *PHO5* and *GAL10*) decreased 2- to 4-fold in the *tra1*${}_{Q3}$ strain. Expression of the SAGA-repressed *ARG1* promoter increased >6-fold. Expression from the *NCW2* promoter was unaltered in the *tra1*${}_{Q3}$ strain.

To examine genetic interactions between *tra1*${}_{Q3}$ and SAGA and NuA4 component genes, the haploid *tra1*${}_{Q3}$ strain was mated with relevant knock-out strains (Winzeler and Davis 1997), and double mutant strains selected after sporulation. Double mutants of *tra1*${}_{Q3}$ with deletions of SAGA component genes *GCN5*, *ADA2*, *SPT3*, *SPT8*, *SPT20*, and *SGF73* and the NuA4 component gene *EAF7* were not obtained suggesting synthetic lethality. Synthetic slow growth on YPD was seen with the SAGA component genes *SGF29*, *SGF11* and *UBP8* and the NuA4 component gene *EAF3* (Figure S5). These genetic interactions resemble those of double deletions of NuA4 and SAGA components (see for example Mitchell *et al.* (2008)).

### Tra1${}_{\mathrm{Q3}}$ is inefficiently transported to the nucleus

**Figure 6:**
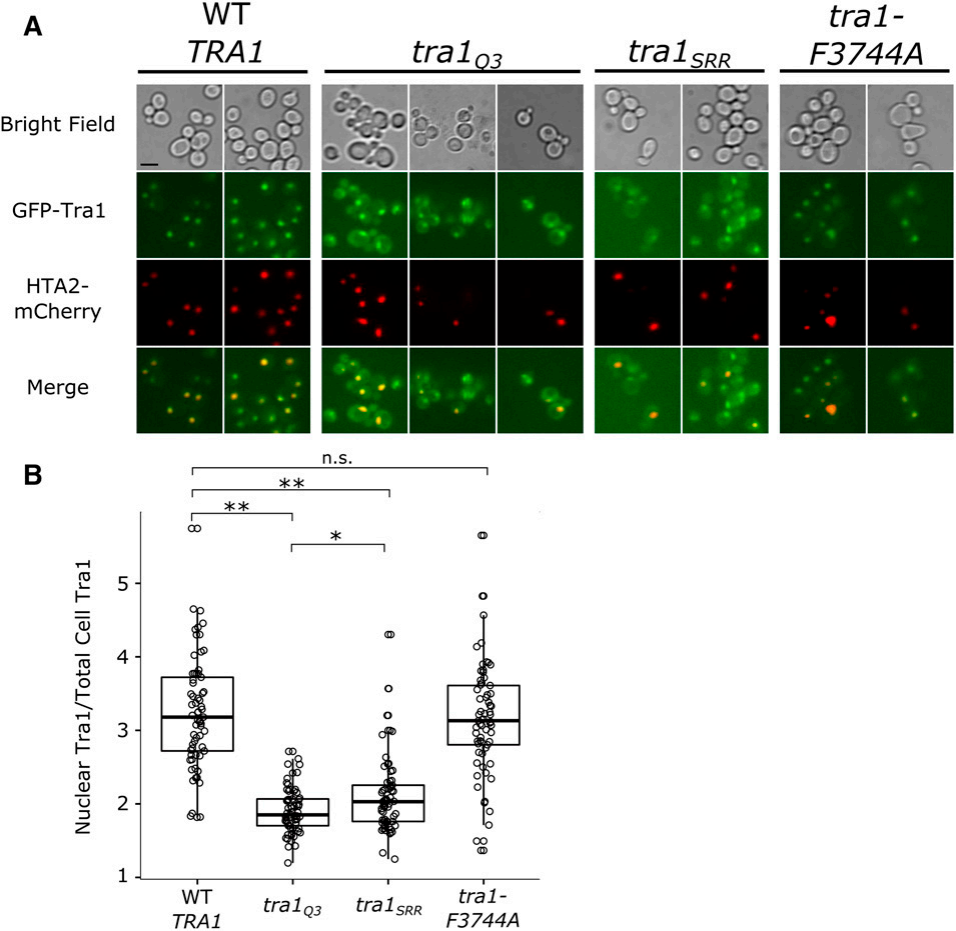
Tra1${}_{\text{Q}3}$ is inefficiently transported to the nucleus. (A) Yeast strains expressing *eGFP-TRA1* (CY5998), *eGFP-tra1${}_{\mathrm{Q3}}$* (CY7341), *eGFP-tra1${}_{SRR}$* (CY7607) or *eGFP-tra1-F3744A* (CY6016) and *HTA2-mCherry* were grown to stationary phase in media lacking uracil and containing 200 *μ*g/mL G418, diluted 1:20 and grown to mid-logarithmic phase. Cells were washed twice in sterile water before imaging. The scale bar represents 5 *μ*m (B) Box-plot showing the ratio of nuclear to cytoplasmic GFP signal from the strains presented in part (A). The box signifies the upper and lower quartiles and the median is represented by the solid black line within the box. Each point represents signal from one individual cell. Signal was quantified using ImageJ for 65 individual cells from each strain. The nucleus was identified using the mCherry signal and the total cell was identified from bright field images. Cytoplasmic signal was calculated as the difference between the total signal and the nuclear signal. Stars indicate statistically significant differences between means according to Welch′s *t*-test and testing at a significance level of *α* = 0.01 after Bonferroni correction (** = *P* < 0.001, * = *P* < 0.01, n.s. = *P* > 0.01).

The stress sensitive phenotype, transcriptional defects and synthetic lethality with SAGA and NuA4 genes exhibited by *tra1*${}_{Q3}$ is similar to other *tra1* PI3K and FATC alleles we have characterized, including *tra1-SRR${}_{3413}$*AAA (*tra1${}_{SRR}$*, an allele with mutations to an alpha helix in the N-terminal lobe of the PI3K domain; Mutiu *et al.* (2007)) and *tra1-F3744A* (an allele with a mutation at the terminal residue of the FATC domain; Hoke *et al.* (2010)). The *tra1${}_{\text{SRR}}$* mutations also fall near the cleft between the N- and C-terminal of the PI3K domain, when mapped onto the recent cryo-EM structure of Tra1 (Figure S6), although it is a less severe allele than *tra1*${}_{Q3}$. To further examine similarities and differences between the expressed proteins, we compared their localization after N-terminal eGFP-tagging (Figure 6A). The ratio of the nuclear GFP-Tra1 to cytoplasmic GFP-Tra1 signal was 5.1 ± 1.4 for the wild-type strain, compared to 2.3 ± 0.4 for the *tra1*${}_{Q3}$ strain (Figure 6B). This result was confirmed with fractionation, where Tra1${}_{\text{Q}3}$ was primarily cytoplasmic and wild-type Tra1 was primarily nuclear (Figure S7). Similar to Tra1${}_{\text{Q}3}$, Tra1${}_{\text{SRR}}$ also mislocalizes to the cytoplasm. In contrast, Tra1-F3744A resembles the wild-type protein with a ratio of 4.6 ± 1.4. We note that Tra1-F3744A does localize to the cytoplasm when cells are grown in media containing ethanol (Genereaux *et al.* 2012), similar to the cytoplasmic localization of Tra1${}_{\text{Q}3}$ and Tra1${}_{\text{SRR}}$ seen in synthetic complete media. We conclude that Tra1${}_{Q3}$ is less efficiently transported to the nucleus than the wild-type protein.

We determined the localization of eGFP-tagged Spt7 in the *tra1*${}_{Q3}$ strain. As shown in Figure S8, eGFP-Spt7 localizes to the nucleus in both wild-type and *tra1*${}_{Q3}$ strains.

**Figure 7:**
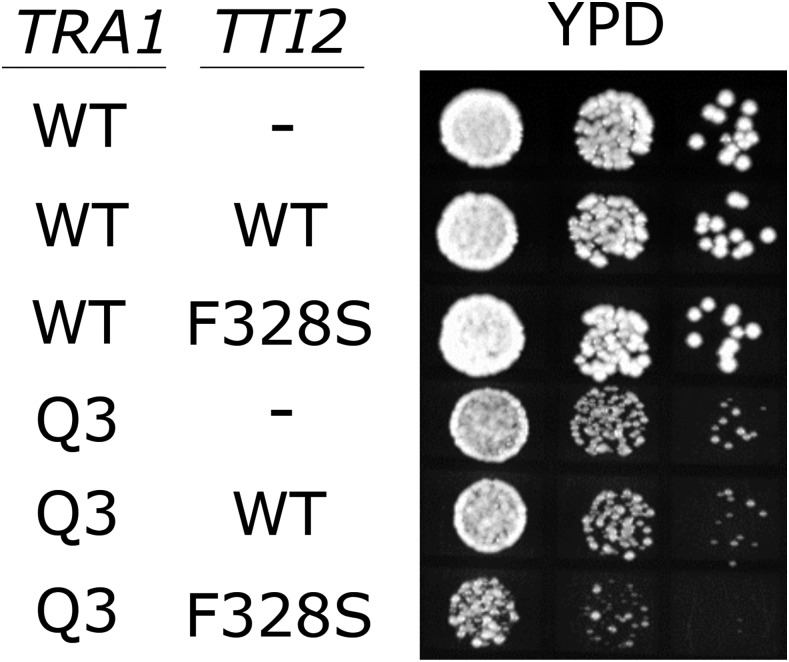
*tti2-F328* is synthetically lethal with *tra1${}_{\mathrm{Q3}}$*. Both *TRA1* (CY4353) and *tra1${}_{\mathrm{Q3}}$* (CY6582) were transformed with either YCplac33 (-), YCplac33-*TTI2* (WT) or YCplac33-*tti2-F328S* (F328S). Strains were grown to saturation in media lacking leucine and uracil, plated in 10-fold serial dilutions on YPD and grown for two days at 30°.

We previously identified a mutation in *TTI2* (*tti2-F328S*) that suppresses slow growth due to *tra1-F3744A* (Genereaux *et al.* 2012). The role of Tti2 as a co-chaperone (Takai *et al.* 2010; Hoffman *et al.* 2016) suggested that Tti2 assists in the folding, stability or quality control of Tra1. As it may further differentiate the PI3K and FATC-mutated *tra1* alleles, we addressed whether the growth defects resulting from *tra1${}_{\mathrm{Q3}}$* could be suppressed by *tti2-F328S*. A diploid strain from a cross of CY6582 (*tra1*${}_{Q3}$*-HIS3*) and CY5665 (*tti2-F328S*) was sporulated. Seven random His+ spore colonies were examined for the nature of the *TTI2* allele. Of the spore colonies containing *tra1*${}_{Q3}$*-HIS3*, none contained *tti2-F328S*, suggesting that *tti2-F328S* is synthetic lethal with *tra1*${}_{Q3}$. To support this conclusion, we transformed *tti2-F328S* on a centromeric plasmid into CY4535 (*TRA1-HIS3*) and CY6582 (*tra1*${}_{Q3}$*-HIS3*). The *tti2-F328S* containing plasmid acted dominantly causing slow growth in the *tra1*${}_{Q3}$ strain (Figure 7). The synthetic lethality with *tti2-F328S*, like the differences in localization, suggests that the defect in Tra1${}_{Q3}$ is distinct from that of Tra1-F3744A.

### Tra1*${}_{\mathrm{Q3}}$* interactions with SAGA and NuA4

**Figure 8:**
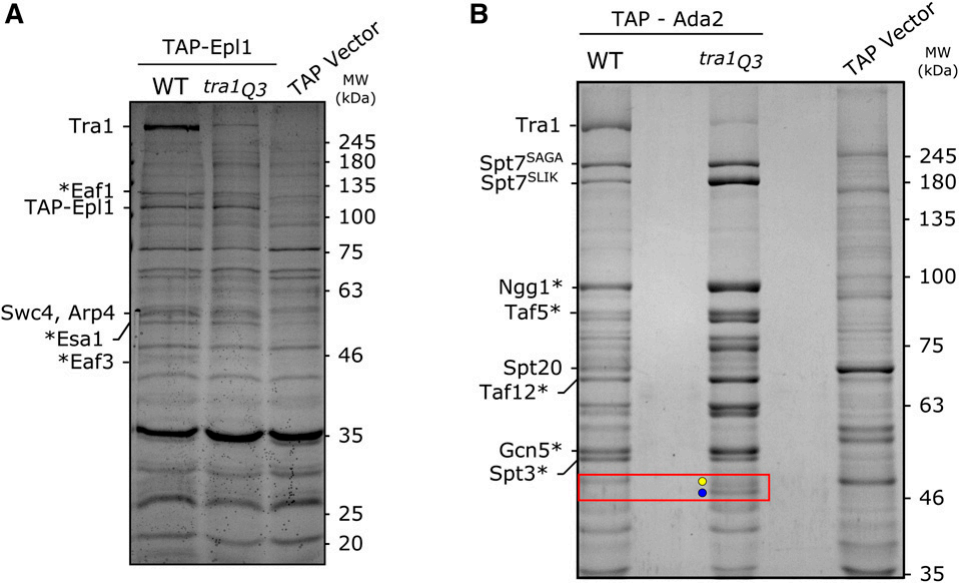
*tra1${}_{\mathrm{Q3}}$* is depleted in pull downs of SAGA and NuA4 components. (A) Strains CY1838 (*TRA1*) and CY7253 (*tra1${}_{\mathrm{Q3}}$*), the containing TAP-tagged Epl1, or BY4741 containing an empty TAP-tag vector (TAP Vector) were grown to an OD${}_{600}$ ∼2.0. 260 mg protein was loaded onto an IgG column and rotated at 4° for two hours. The column was washed and protein cleaved from the column using TEV protease before chromatography on a calmodulin column. Eluted protein was precipitated, resuspended in sample buffer and separated by SDS-PAGE (10%). The gel was stained with CBB. Labeled bands were identified using mass spectrometry or predicted based on mass and previous pull downs (Steunou *et al.* (2016); indicated by *). (B) CY1775 (*TRA1*) and CY7042 (*tra1${}_{\mathrm{Q3}}$*), containing TAP-tagged Ada2, or BY4741 containing an empty TAP-tag vector (TAP Vector) were grown to an OD${}_{600}$ ∼2.0. 170 mg protein was used in the pull down following the above protocol. Protein was separated by SDS-PAGE (8%).Labeled bands were identified using mass spectrometry or predicted based on mass and previous pull downs (Grant *et al.* (1998); indicated by *). A red box highlights the proteolytic fragments of Spt20 in the *tra1${}_{\mathrm{Q3}}$* strain.

Considering the increased cytoplasmic Tra1${}_{Q3}$ relative to wild-type Tra1, we hypothesized that the association of Tra1${}_{Q3}$ with the SAGA and NuA4 complexes may be altered. Using TAP-tagged Ada2 and Epl1, we pulled down SAGA and NuA4, respectively, in strains containing either wild-type *TRA1* or *tra1*${}_{Q3}$ as the sole copy. Interacting proteins were separated by SDS-PAGE and visualized by staining with Coomassie Brilliant Blue (Figure 8). Most apparent in the Epl1-NuA4 pull down was the decreased association with Tra1${}_{Q3}$. From two biological replicates, we found a 6.7 ± 1.5 -fold decrease in the amount of Tra1${}_{Q3}$ associating with Tap-Epl1 compared to wild-type Tra1. Swc4 and Arp4, migrating at ∼60 kD and ∼42 kD, respectively, were unchanged in wild-type and *tra1*${}_{Q3}$ pull downs.

The TAP-Ada2 pull-down showed that Tra1${}_{Q3}$ also associates less efficiently than wild-type Tra1 with components of the SAGA complex (Figure 8B). Assuming equal loading, we quantified a 6.5 ± 0.3-fold decrease in Tra1${}_{Q3}$ compared to wild-type Tra1 from the average of three biological replicates. In addition to decreased association of Tra1${}_{Q3}$ with SAGA, other changes in the pattern of interacting proteins were noted in the TAP-Ada2 pull down. Full-length Spt7 (Spt7^SAGA^) and the truncated SLIK form of Spt7 (Spt7^SLIK^) migrate with predicted molecular masses of 245 kD and 180 KDa, respectively. From the average of three biological replicates, the ratio of Spt7^SAGA^ to Spt7^SLIK^in the wild-type strain was 2.5 ± 0.6, whereas in the *tra1*${}_{Q3}$ strain the ratio was 0.5 ± 0.1. Using myc^9^-tagged Spt7, we confirmed the relative increase of Spt7^SLIK^ in the *tra1*${}_{Q3}$ by western blotting (Spt7^SAGA^/Spt7^SLIK^ in the wild-type strain was ∼4.5, whereas in the *tra1*${}_{Q3}$ it was ∼1.7; Figure S9).

**Figure 9:**
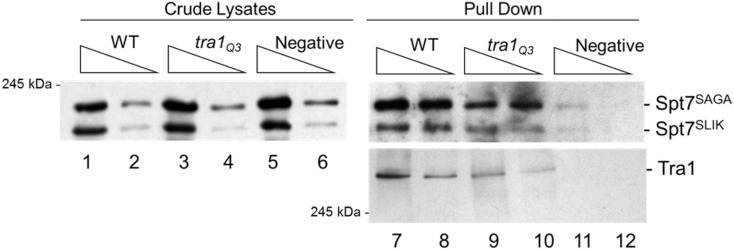
Spt7^SLIK^ increases due to stress caused by *tra1${}_{\mathrm{Q3}}$*. Diploid strains CY7405 (*Flag*^5^*-TRA1/TRA1*), CY7406 (*Flag*^5^*-tra1${}_{\mathrm{Q3}}$*/TRA1) and CY7407 (*TRA1/TRA1*) containing myc^9^-*SPT7* on a *URA3* centromeric plasmid were grown to an OD${}_{600}$ ∼2.0 and protein harvested by grinding in liquid nitrogen. Tra1 was pulled down using anti-Flag M2 magnetic beads from 2 mg of crude lysate. Protein was separated by SDS-PAGE (5%). Spt7 and Tra1 were detected with anti-myc and anti-Flag antibodies respectively.

As SLIK plays a role in response to stress, the increased SLIK form of Spt7 in the Tra1${}_{Q3}$ strain could be a consequence of stress resulting from the *tra1*${}_{Q3}$ mutation. To evaluate this possibility, we created heterozygous strains containing *TRA1/Flag*^5^*-TRA1* or *TRA1/Flag*^5^*-tra1*${}_{Q3}$. We pulled down tagged Tra1 using anti-Flag antibody and blotted for association of myc^9^-tagged Spt7. In this case the ratio of Spt7^SAGA^ to Spt7^SLIK^ associating with wild-type Tra1 and Tra1${}_{Q3}$ was similar (Figure 9). We thus conclude that the altered ratio of full-length Spt7 to the SLIK form of Spt7 in the haploid *tra1*${}_{Q3}$ strain is due to inducing stress rather than an inherent change in the ability of the Tra1${}_{Q3}$ protein to interact with either form.

**Figure 10:**
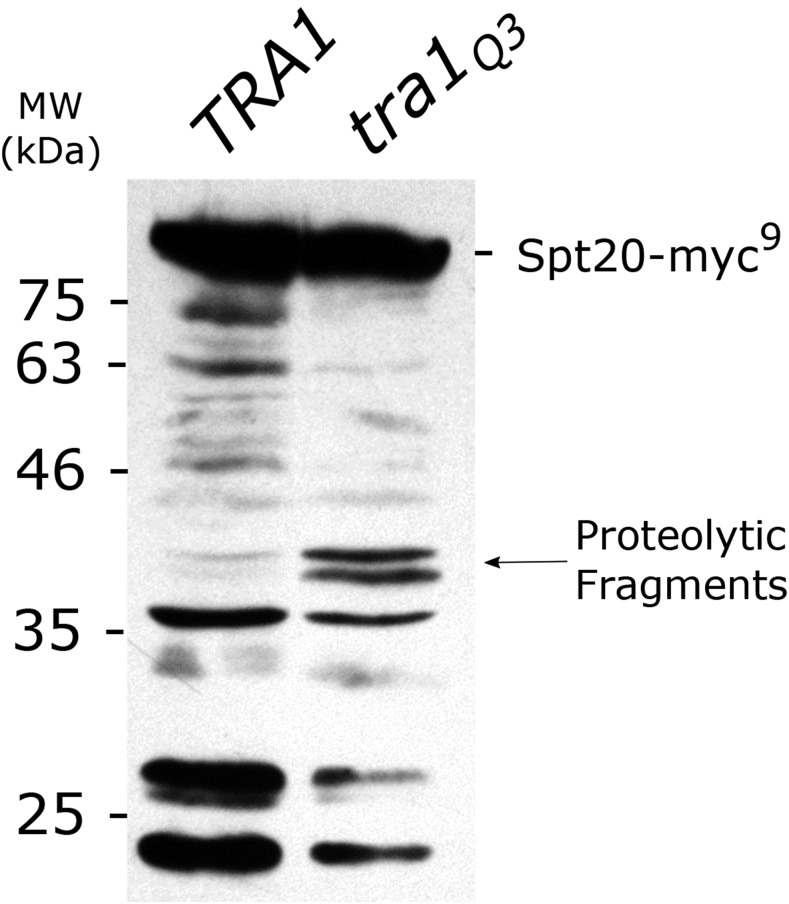
Spt20 is degraded in the presence of *tra1${}_{\mathrm{Q3}}$*. Strains CY7530 (*TRA1*) or CY7531 (*tra1${}_{\mathrm{Q3}}$*) containing *TAP-ADA2* and YCplac33-*SPT20-myc*^9^ were grown to saturation in media lacking uracil, diluted 1:10 in YPD and grown for eight hours. Cells were harvested and lysed using glass beads. 200 *μ*g protein was separated by SDS-PAGE (10%), transferred to a PVDF membrane and blotted with an anti-myc antibody.

A second difference between wild-type Tra1 and Tra1${}_{Q3}$ in the TAP-Ada2 pull down (Figure 8B) was altered association with Spt20. From two biological replicates, the amount of full length Spt20 decreased 2.1 ± 0.7-fold in the *tra1*${}_{Q3}$ strain relative to the wild-type. Corresponding to this, two Spt20 proteolytic products of ∼46 kDa appeared. Mass spectrometry of the truncated proteins identified consistent coverage of Spt20 peptides to residue 485, suggesting that the N-terminal portion of Spt20 remains associated with SAGA in the *tra1*${}_{Q3}$ background (Figure S10). To confirm the presence of an Spt20 proteolytic fragment in the *tra1*${}_{Q3}$ strain, we C-terminally tagged *SPT20* with a myc epitope and blotted for the protein in wild-type and *tra1*${}_{Q3}$ strains. We note that an N-terminally tagged *SPT20* molecule does not complement an *spt20* deletion (Figure S11). In agreement with the appearance of proteolytic products of Spt20 in the Coomassie Brilliant Blue stained pull down, two bands migrating at ∼37 kDa were enhanced in the *tra1*${}_{Q3}$ strain crude lysates (Figure 10). Since Han *et al.* (2014) demonstrated contacts between Tra1 and Spt20, this result is consistent with Tra1${}_{Q3}$ associating poorly with SAGA and thus Spt20 being susceptible to proteolytic cleavage.

### Intragenic suppressors of tra1${}_{\mathrm{Q3}}$ restore localization

**Figure 11:**
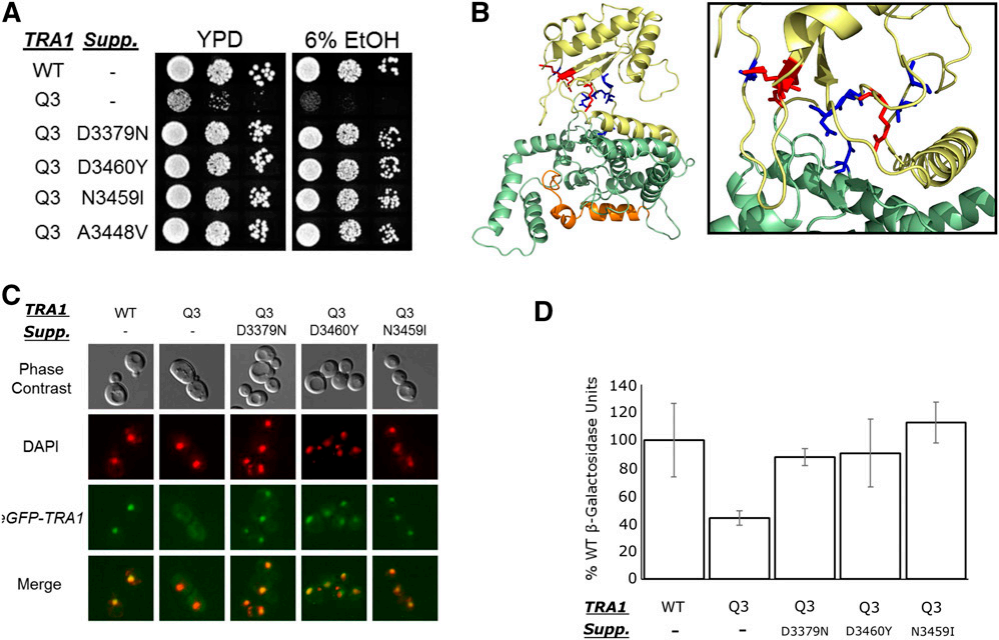
Second site suppressors of *tra1${}_{\mathrm{Q3}}$*. (A) Second site suppressors rescue slow growth of *tra1${}_{\mathrm{Q3}}$*. Yeast strains CY4353 (*TRA1*), CY6582 (*tra1${}_{\mathrm{Q3}}$*) and four independent suppressors that restore growth of *tra1${}_{\mathrm{Q3}}$* on media containing ethanol were grown to stationary phase in media lacking leucine to maintain YCplac111-*YHR100C*, diluted in 10-fold dilutions and spotted onto YPD or YPD containing 6% ethanol and grown at 30° for two days. (B) Intragenic suppressors were mapped onto the cryo-EM Tra1 structure (Diaz-Santin *et al.* 2017). The three initial arginine to glutamine mutations are highlighted in red. Residues that suppressed *tra1${}_{\mathrm{Q3}}$* are blue. (C) Second site suppressors restore nuclear localization of Tra1${}_{\text{Q}3}$. An N-terminal eGFP tag was integrated in front of four *tra1${}_{\mathrm{Q3}}$* suppressor alleles. Yeast strains CY5998 (*eGFP-TRA1*), CY7341 (*eGFP-tra1${}_{\mathrm{Q3}}$*) and the GFP tagged suppressors were grown to stationary phase in media lacking uracil, diluted 1:20 and grown to mid-logarithmic phase and stained with DAPI. Cells were washed twice with water before imaging. (D) Second site suppressors restore transcription defects of *tra1${}_{\mathrm{Q3}}$*. Strains BY4741 (*TRA1*), CY6635 (*tra1${}_{\mathrm{Q3}}$*) and suppressor strains CY6909 (*tra1${}_{\mathrm{Q3}}$*-D3379N), CY6902 (*tra1${}_{\mathrm{Q3}}$*-D3460Y), and CY6940 (*tra1${}_{\mathrm{Q3}}$*-N3459I) were transformed with SRE-*lacZ* on a *LEU2* centromeric plasmid. Strains were grown to saturation in media lacking leucine before being diluted 1:10 in YPD with 6% ethanol grown for eight hours. *β*-galactosidase activity units are the average of three replicates with the SD shown by the error bars.

Tra1${}_{Q3}$ has decreased nuclear localization and results in a slow growth phenotype. The altered localization could be responsible for the phenotypic changes or result from the phenotypic changes. We sought to identify second site mutations that would suppress the phenotypes to differentiate between these possibilities. We selected for spontaneous mutations and for mutations induced by UV irradiation or treatment with sodium azide that suppress the slow growth of CY6582 (*tra1${}_{\mathrm{Q3}}$*) on media containing 6% ethanol (Figure 11A). All suppressor strains also grew on media containing Calcoflour white and at high temperature. To determine if the suppressor mutations were intragenic or extragenic, the strains were crossed with a wild-type strain and the segregation of the suppressor mutation relative to the *tra1${}_{\mathrm{Q3}}$* allele determined after sporulation. In all cases, the suppressor mutation segregated with *tra1${}_{\mathrm{Q3}}$*, indicating that they were intragenic. We identified revertants back to arginine at all three mutated glutamine residues. In addition to the arginine revertant at position 3389, lysine and histidine acted as suppressors, stressing the importance of the positive charge at that position. Six additional mutations at five different positions within the PI3K domain suppressed the slow growth of *tra1${}_{\mathrm{Q3}}$* (Table 1). When mapped to the cryo-EM Tra1 structure (Diaz-Santin *et al.* 2017), these residues fall within the cleft of the kinase domain, with the exception of A3448V (Figure 11B).

**Table 1:** Suppressor residues that reverse the slow growth phenotype of *tra1${}_{\mathrm{Q3}}$*

Residue #	Residue in *tra1${}_{\mathrm{Q3}}$*	Suppressor Residue(s)
3379	D	N,Y
3448	A	V
3450	P	S
3459	N	I
3460	D	Y
3580	N	S
3389	Q	R (Revertant), K, H
3390	Q	R (Revertant)
3456	Q	R (Revertant)

The intragenic suppressors allowed us to assess whether suppression of the slow growth phenotype correlated with the localization of *tra1${}_{\mathrm{Q3}}$*. As shown in Figure 11C, suppressor mutations D3379N, D3460Y, and N3459I restored nuclear localization of Tra1 in the context of the triple mutations. The suppressors also restored the half-life of Tra1${}_{Q3}$ to greater than 8 hr (Figure S12). It is thus likely the growth defects observed in the *tra1${}_{\mathrm{Q3}}$* strain are due to inefficient nuclear transport of Tra1${}_{Q3}$ and related to the nuclear functions of Tra1. We also evaluated the three suppressor mutations to determine if they restore transcription of SRE-*lacZ* fusion in the *tra1${}_{\mathrm{Q3}}$* background. Suppressor mutations D3379N, D3460Y or N3459I restored transcription to near wild-type levels (Figure 11D) suggesting that the *tra1${}_{\mathrm{Q3}}$* phenotypes are due to defects in transcription.

## Discussion

### The Tra1 cleft region is important for function

It is difficult to predict the exact structure of the Tra1 kinase cleft, as there are at least two locations in the cleft where gaps are introduced when aligning the sequences of Tra1 with other PIKK proteins (Figure S2) and the cryo-EM structure is at 3.7 Å resolution. Three arginine residues within or proximal to the cleft (R3389, R3390 and R3456) stood out due to their conservation in Tra1 orthologs and potential to be solvent exposed. Indicative of their importance, mutating these arginine residues to glutamine resulted in transcriptional changes, slow growth and reduced the efficiency of transport to the nucleus. Tra1${}_{\text{A}3}$, with alanine at the same positions, would not support viability. In contrast to Tra1${}_{\text{Q}3}$, altering R3456 alone to glutamine or R3389 and R3390 as a pair had a modest effect, suggesting possible redundancy in the role of the positive charge.

Intragenic suppressor mutations of *tra1${}_{\mathrm{Q3}}$* that mapped to the cleft region also support the importance of residues within and proximal to the cleft. The genetic network comprised of the *tra1${}_{\mathrm{Q3}}$* mutations and the suppressor mutations outline a possible Tra1 PI3K binding cleft and will support current and future biophysical analyses of structure.

### The cleft mutations of Tra1${}_{\mathrm{Q3}}$ affect nuclear localization and assembly into SAGA and NuA4 complexes

Tra1${}_{\text{Q}3}$ associated poorly with SAGA and NuA4 and gave rise to phenotypes characteristic of deletions in other components of these complexes. Many if not all of the functions of SAGA and NuA4 likely require Tra1 since these phenotypes arise in the *tra1${}_{\mathrm{Q3}}$* strain, where SAGA and NuA4 are otherwise relatively intact. For chromatin targets, this is probably due to Tra1’s role in recruiting SAGA and NuA4 to promoters.

The size of Tra1 demands that it be actively imported into the nucleus. Tra1${}_{\text{Q}3}$ was more prevalent in the cytoplasm raising the question of whether lack of assembly into SAGA/NuA4 is causing defective localization or whether defective localization is causing lack of assembly. We favor the idea that association is required for nuclear localization because Tra1 is devoid of obvious nuclear localization signals. We speculate that Tra1 “piggy-backs” on another SAGA and/or NuA4 component for entry into the nucleus in partially or completely assembled complexes. This is consistent with work done by Kassem *et al.* (2016), which identified the co-translational assembly of Ada2, Gcn5 and Spt20 and that proper co-translational assembly is required for Gcn5 import into the nucleus. An analogy is RNA polymerase II (RNAPII), a 12 subunit complex whose assembly into subcomplexes occurs in the cytoplasm with the help of chaperones (Boulon *et al.* 2010; Wild and Cramer 2012). The RNAPII subcomplexes are then imported into the nucleus (Czeko *et al.* 2011). When interactions required for assembly are disrupted, RNAPII accumulates in the cytoplasm (Forget *et al.* 2010). In a similar way, our work supports a model whereby Tra1 is incorporated into SAGA/NuA4 in the cytoplasm, creating either a complete complex or partial complexes that are then imported into the nucleus. While Tra1${}_{\text{Q}3}$ is poorly transported to the nucleus and fails to associate with SAGA, Spt7 remains associated with SAGA and is nuclear in the *tra1${}_{\mathrm{Q3}}$* strain. This suggests nuclear import of Spt7 does not require Tra1 incorporation into SAGA.

### A feedback mechanism regulates expression of SAGA and NuA4 components

The steady state level of Tra1${}_{\text{Q}3}$ was slightly elevated relative to the wild-type protein yet its half-life was reduced threefold. As the alleles analyzed in this study were expressed in their native context, the decreased half-life suggested that reduced levels of functional Tra1 trigger increased transcription from the *TRA1* promoter. A transcriptional feedback mechanism was confirmed both with RT-qPCR and a *TRA1-lacZ* promoter fusion, where expression increased ∼threefold in the *tra1${}_{\mathrm{Q3}}$* background. Interestingly, the regulation appears to be mediated only via Tra1 and not through depletion of other SAGA or NuA4 components. Further studies will be required to determine the mechanism of this regulation and to investigate if other SAGA/NuA4 genes are coordinately regulated.

We envisage two mechanisms that could account for the reduced half-life of Tra1${}_{\text{Q}3}$. It is possible that the mutated protein is inherently unstable. Arginine to glutamine changes were made to reduce this possibility, and the residues appear to be surface exposed; however, the mutations could indirectly alter the conformation of another region of the protein that then leads to a reduced half-life. Alternatively, the reduced stability may be the consequence of the glutamine mutations, directly or indirectly, reducing the assembly of Tra1 into complexes, leaving the 3744-residue protein accessible to proteolytic cleavage.

### Effects of Tra1${}_{\mathrm{Q3}}$ on the composition of SAGA and NuA4 complexes

The size of Tra1 often leads to the suggestion that it scaffolds the formation of SAGA and NuA4. However, Helmlinger *et al.* (2011) showed in *Schizosaccharomyces pombe* that when the non-essential paralog Tra1 is deleted, SAGA assembles normally. Our experiments support the idea that Tra1 is not required to form the complexes in *S. cerevisiae*. Robust association of SAGA and NuA4 components with TAP-Ada2 and TAP-Epl1, respectively were observed in the *tra1${}_{\mathrm{Q3}}$* background, where there is reduced association of Tra1. These complexes were maintained through both extract preparation and affinity purification. We were unable to identify all complex components in the pull-down so we cannot conclude definitively that Tra1 does not play a role in the association of some components. However its peripheral localization in the structures of both complexes and the limited number of crosslinks between Tra1 and SAGA components suggest that this is unlikely (Wu *et al.* 2004; Chittuluru *et al.* 2011; Han *et al.* 2014). Spt20 crosslinks to the FAT domain of Tra1 (Han *et al.* 2014) indicating the close proximity of these proteins. Our finding that Spt20 is proteolytically cleaved in the *tra1${}_{\mathrm{Q3}}$* background is further evidence for their proximity. N-terminal fragments of Spt20 that remove approximately 200 residues remained associated with SAGA, while a C-terminal fragment that migrated with an estimated molecular weight of approximately 37 kD was found in whole cell extracts. This suggests that the C-terminal fragment of Spt20 is not required for the association of Spt20 with SAGA. We believe that the Spt20 fragments are derived after cell lysis and do not play a physiological role because the Spt20 fragments are not apparent if cells are rapidly lysed and proteins denatured with alkali. We note that Kassem *et al.* (2016) observed Spt20 fragments associating with SAGA when SAGA improperly assembled. By analogy, the proteolytic fragments of Spt20 seen in the *tra1${}_{\mathrm{Q3}}$* strain is consistent with a reduced ability of Tra1${}_{\text{Q}3}$ to assemble into SAGA.

### Possible functions of the Tra1 pseudokinase

Approximately 10% of kinases are predicted to be pseudokinases due to absence of at least one of the required kinase motifs (Boudeau *et al.* 2006). One common role for pseudokinases is to allosterically activate other enzymes. For example, the receptor tyrosine pseudokinase HER3 upon ligand binding, interacts with HER2 resulting in HER2 autophosphorylation and activation (Holbro *et al.* 2003). Similarly the pseudokinase STRAD*α* binds to LKB1 allowing translocation to the cytoplasm and G1 cell cycle arrest (Baas *et al.* 2003). Pseudokinase domains are also found in proteins containing active kinase domains where they play a regulatory role. For example the pseudokinase domain of the Janus kinase (JAK) family proteins inhibits the kinase domain in the absence of stimulus (Lupardus *et al.* 2014; Brooks *et al.* 2014). Like kinases, pseudokinases can undergo a conformational change that is required for function (*e.g.*, STRAD*α*; Zeqiraj *et al.* (2009b, a)). Small molecules may induce the conformational change, as is seen in the binding of 2′,5′-oligoadenylate to the pocket between the ankyrin and pseudokinase domain of RNase L (Dong and Silverman 1999).

We speculate that the PI3K pseudokinase domain of Tra1 regulates the association of Tra1 with other components of SAGA and NuA4 through an allosterically regulated mechanism. The fact that in the kinase members of the family, the cleft region of the PI3K domain binds both a small molecule and a protein substrate leads us to suggest that it may also bind a ligand in Tra1. The putative surface location of the mutated arginine residues in Tra1${}_{\text{Q}3}$ and their ionic character is consistent with a molecular interaction. The increased hydrophobicity of many of the amino acid residue changes of the suppressor mutations (D3379Y, A3348V, N3459I, and D3460Y) would decrease the dielectric constant within the cleft (Perutz 1967), which could strengthen the remaining ionic interactions between Tra1 and a charged ligand. The interaction could promote a conformational change in Tra1 that facilitates interaction directly with SAGA or NuA4, enhance Tra1 half-life thus improving the kinetics of association or alter the precise localization of Tra1.

We can only speculate on the nature of any PI3K domain interacting molecule. It is unlikely that the ligand is a component of SAGA because Han *et al.* (2014) did not find SAGA components crosslinking with the PI3K domain of Tra1. Given the importance of the positive charge in the cleft region, it is interesting to consider some candidate small molecule ligands that carry negative charge. These include inositol phosphates, inositol pyrophosphates, and phosphatidylinositols. Each is attractive because of their regulatory roles and involvement in gene expression (*e.g.*, Streb *et al.* (1983); Mellman *et al.* (2008); Watson *et al.* (2012); Wilson *et al.* (2013). We note that while we find that Ada2 and Spt7 bind phosphatidylinositol (Hoke *et al.* 2008a), we have been unable to demonstrate a similar interaction with the recombinant PI3K domain of Tra1. The list of other potential ligands is large since more than 200 metabolic and biosynthetic intermediate molecules are phosphorylated in yeast (Ramirez-Gaona *et al.* 2017), including phosphorylated intermediates in amino acid biosynthesis, in lipid biosynthesis, Kreb cycle intermediates and even sugar phosphates. We have also considered the binding of a small RNAs to the PI3K domain of Tra1 though we have not found RNA to copurify with SAGA. We initiated the genetic suppression studies with *tra1${}_{\mathrm{Q3}}$* with the goal of providing clues to a binding partner for the PI3K domain of Tra1. Unfortunately, no extragenic suppressors were identified in what we consider to be extensive screening. The nature of the molecular interactions of the PI3K domain of Tra1 are thus elusive and will likely require a detailed metabolomic analysis in native cells.
